# Clinicopathological features and BRAF^V600E^ mutations in patients with isolated hypothalamic-pituitary Langerhans cell histiocytosis

**DOI:** 10.1186/s13000-016-0548-5

**Published:** 2016-10-19

**Authors:** Zhen Huo, Tao Lu, Zhiyong Liang, Fan Ping, Jie Shen, Jingjing Lu, Wenbing Ma, Dachun Zhao, Dingrong Zhong

**Affiliations:** 1 Department of Pathology, Peking Union Medical College Hospital, Chinese Academy of Medical Sciences & Peking Union Medical College, No.1 Shuaifuyuan, Wangfujing Street, Dongcheng District, Beijing, 100730 China; 2 Department of Endocrine, Peking Union Medical College Hospital, Chinese Academy of Medical Sciences & Peking Union Medical College, Beijing, 100730 China; 3 Department of Radiation Oncology, Peking Union Medical College Hospital, Chinese Academy of Medical Sciences & Peking Union Medical College, BeiJing, 100730 China; 4 Department of Radiology, Peking Union Medical College Hospital, Chinese Academy of Medical Sciences & Peking Union Medical College, Beijing, 100730 China; 5 Department of Cerebral Surgery, Peking Union Medical College Hospital, Chinese Academy of Medical Sciences & Peking Union Medical College, BeiJing, 100730 China

**Keywords:** Langerhans cell histiocytosis, Hypothalamic-pituitary, Central diabetes insipidus, Anterior pituitary function, BRAF mutation

## Abstract

**Background:**

Isolated hypothalamic-pituitary Langerhans cell histiocytosis (HPLCH) is very rare. We investigated the clinicopathological characteristics, endocrine function changes, BRAF^V600E^ mutations and treatments of isolated HPLCH.

**Methods:**

We identified seven patients with isolated HPLCH by reviewing the clinical and pathological files in our hospital from 2007 to 2015. The clinical characteristics of the seven patients were retrospectively reviewed, especially the endocrine function changes. Immunostaining and mutation profiling of BRAF^V600E^ were performed.

**Results:**

The seven HPLCH patients included three men and four women, aged 9–47 years. All patients presented with symptoms of central diabetes insipidus (CDI), and four displayed anterior pituitary hypofunction as well. Magnetic resonance imaging showed hypothalamic-pituitary axis involvement in all patients. There was no evidence for the involvement of other organs in all seven patients. Langerhans cell histiocytosis was confirmed by neuroendoscopic procedures, and immunohistochemical staining showed that all cases (7/7) were positive for CD68, CD1a, Langerin, and S-100. The BRAF^V600E^ mutation was detected in three of the six cases (3/6). Six patients had follow-up information; all received desmopressin acetate and high-dose corticosteroid therapy, and two patients received radiotherapy.

**Conclusions:**

Our study indicated that all patients with isolated HPLCH had CDI as the earliest symptom, and more than half of the patients had anterior pituitary deficiencies. The BRAF^V600E^ mutation is a common genetic change in HPLCH patients. Treatment of HPLCH patients is difficult, and the progressive loss of endocrine function is irreversible in most cases.

## Background

Langerhans cell histiocytosis (LCH) is characterized by the idiopathic proliferation of specialized bone marrow-derived Langerhans cells and mature eosinophils. LCH can affect any organ or system and may be systemic or localized [1, 2]. Patients with isolated hypothalamic-pituitary (HP) LCH are very rare, although patients with multisystemic LCH often show pituitary gland involvement [3–7]. Among the endocrine regions, LCH is frequently found in the HP region, resulting in diabetes insipidus (DI), the most common endocrine anomaly [8–12]. Anterior pituitary involvement also occurs as a result of the disease process. However, anterior pituitary dysfunction is not invariably associated with abnormal HP region imaging, and it is almost always encountered in patients with multisystemic disease who show DI and HP pathology on magnetic resonance imaging (MRI) [6, 8]. The anterior pituitary endocrine function changes in isolated LCH limited to the HP region have been poorly studied.

Recently, LCH patients were shown to have a high frequency of BRAF^V600E^ mutations and to respond to RAF inhibitors, suggesting that LCH is more likely a neoplastic than a reactive disorder. The BRAF^V600E^ mutations are present in approximately 25–60 % of LCH cases [13–20], but this mutation has not been reported in isolated HPLCH in previous publications.

Several studies on LCH patients, which examined relatively small numbers of patients, have provided information on the evolution of pituitary dysfunction as well as the morphological changes in the HP region [3–7]. However, no large studies have examined patients with isolated HPLCH and assessed the pituitary function without interference from other organs. In the current study, we retrospectively studied seven patients with isolated HPLCH in our hospital from 2007 to 2015 and analysed their clinical and pathological features, endocrine function changes, BRAF^V600E^ mutations, and treatment outcomes.

## Methods

### Patients and specimens

We reviewed all surgical biopsy or resection records in the Peking Union Medical College Hospital from January 1, 2007 to December 31, 2015 and identified a total of seven cases with isolated HPLCH. The patients’ medical records, including patient complaints, brain MRI findings, evaluation of anterior pituitary function, evaluation of other organs, and treatment, were collected and reviewed. No patient had a history of LCH. The HP regions of the patients were evaluated by MRI scans before and after treatment. The pre-treatment basal levels of growth hormone (GH), insulin-like growth factor-1 (IGF1), adrenocorticotropic hormone (ACTH), cortisol, free thyroxine (FT4), thyroid-stimulating hormone (TSH), prolactin (PRL), luteinizing hormone (LH), follicle-stimulating hormone (FSH), oestradiol, and testosterone were measured early in the morning in conjunction with plasma and urine osmolality tests. A water deprivation test was performed to assess vasopressin deficiency. In four patients with suspected pituitary dysfunction, dynamic pituitary function tests were employed, including the insulin tolerance test for assessments of GH and/or ACTH/cortisol reserves, thyrotropin-releasing hormone and gonadotropin-releasing hormone stimulation tests, and insulin-induced hypoglycaemia test. The pituitary functions of the patients were evaluated endocrinologically at least once per year. Methods used to evaluate other organs included a B ultrasound, computed tomography (CT), full-body bone scans, and a bone marrow biopsy. In addition, two patients received positron-emission tomography/computerized tomography (PET/CT). All patients received endoscopic transnasal transsphenoidal biopsy or resection of the HP regions. All samples were fixed in 10 % neutral buffered formalin, routinely processed, and embedded in paraffin. Haematoxylin-eosin-stained sections were observed using optical microscopy and reviewed independently by three experienced pathologists. The pathological diagnosis of LCH was based on the World Health Organization (WHO) criteria for LCH [1, 2]. We collected follow-up data from outpatient follow-ups.

### Immunostaining

Immunostaining of CD1a (EP3622, 1:200; ZSJQ-BIO, Beijing, China), S-100 (polyclonal, prediluted; ZSJQ-BIO, Beijing, China), Langerin (12D6, prediluted; ZSJQ-BIO, Beijing, China), and CD68 (KP-1, 1:100; Dako, Glostrup, Denmark) was performed for all seven cases according to the manufacturer’s instructions. Immunostaining was performed on 4 μm-thick unstained sections cut from representative formalin-fixed paraffin-embedded (FFPE) blocks. For all markers, positive controls and negative controls were used. For CD68, signals appearing as tan particles in the cytoplasm were considered positive. For S-100, tan particles in the nucleus or cytoplasm were considered positive. For CD1a, tan particles in the membrane were considered positive. For Langerin, tan particles in the membrane or cytoplasm were considered positive.

### Detection of BRAF^V600E^ mutations

Genomic DNA from six cases of HPLCH (the tissue of the last case was not sufficient for further study) was extracted from freshly cut FFPE tissue sections using a QIAamp DNA Mini Kit (Qiagen, Germany) according to the manufacturer’s instructions. The area of the highest histiocyte density was identified through haematoxylin-eosin staining, and tissue from this area in unstained sections was scraped for DNA extraction. The extracted DNA was then quantified using a Qubit dsDNA BR assay (Life Technologies, USA). DNA from all six cases of LCH was successfully amplified. Analysis of BRAF^V600E^ mutations was performed with a BRAF V600E Mutation Detection Kit (Amoy Diagnostics Co., Ltd., China) according to the manufacturer’s instructions. Real-time quantitative polymerase chain reaction (QPCR) was carried out on an Applied Biosystems7500 QPCR Platform (Life Technologies, USA). The cycling conditions for quality control (QC) runs and for mutation assays were as follows: 5 min incubation at 95 °C, followed by 15 cycles of 95 °C for 25 s, 64 °C for 20 s, and 72 °C for 20 s, and finally 31 cycles of 95 °C for 25 s, 60 °C for 35 s, and 72 °C for 20 s. Fluorescence was measured at 60 °C. Mutation data were interpreted according to the kit manual after curve analysis and calculation of ΔCt values.

## Results

### Clinical data

The seven cases of isolated HPLCH (Table 1) included three men and four women, aged 9–47 years. All seven patients had symptoms before the diagnosis, and the major complaint was polyuria and polydipsia (7/7). In addition, one female patient had secondary amenorrhoea (1/7), and one child patient showed growth retardation (1/7). No patients had visual field defects. All patients had no history of the smoking and drinking. The interval between onset of symptoms and diagnosis was 1–108 months. Before the LCH diagnosis by pathology, all seven patients had been diagnosed with central diabetes insipidus (CDI), and a mass was found in the HP region by MRI. Chest CT scans, abdominal B ultrasound, and full-body bone scans were performed in all seven cases, but no positive findings were obtained. Two patients received PET/CT scans, and there were no positive findings except in the HP region.

**Table 1:** Clinical profile and follow-up data of seven patients with hypothalamic-pituitary Langerhans cell histiocytosis

No.	Age (years)/ Gender	The interval between onset of symptoms and diagnosis	CDI	Sites of involvement		Follow-up	Therapy
		(months)		At presentation	Follow-up		
1	9 / Female	108	Present	PS	None	Alive, 39 ms	RT + HRT
2	47 / Female	1	Present	PS	PS	Alive, 5 ms	HRT
3	12 / Female	36	Present	PS + H	PS + H	Alive, 5 ms	HRT
4	22 / Male	2	Present	PS + P	NA	NA	NA
5	13 / Male	4	Present	PS + H	None	Alive, 41 ms	RT + HRT
6	15 / Male	1	Present	PS + P	PS + P	Alive, 2 ms	HRT
7	35 / Female	18	Present	PS	Liver involvement suspected	Died, 74 ms	RT + Surgery + HRT

*PS* pituitary stalk, *H* hypothalamus, *P* pituitary, *NA* not available, *RT* radiotherapy, *CDI* central diabetes insipidus, *HRT* hormone replacement therapy

**Fig. 1:**
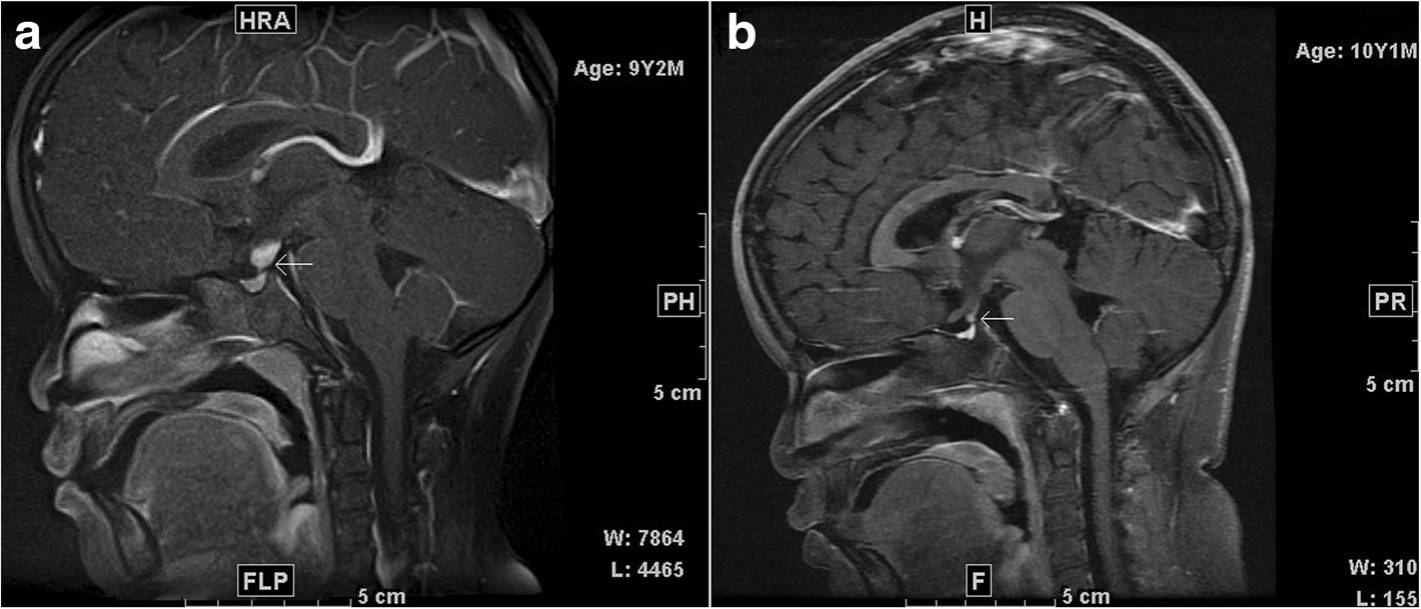
**a**. MRI scan of the lesion (*arrow*) in a 9-year-old child (case 1) before diagnosis demonstrated that the upper pituitary stalk was thickened, and a well-circumscribed and regular homogeneous enhanced mass was observed (sagittal T1 contrast-enhanced images). **b**. MRI scan of the lesion in case 1 at 11 months of follow-up demonstrated that the pituitary stalk (*arrow*) was fine and the homogeneous enhanced mass had regressed, but the anterior pituitary narrowed (sagittal T1 contrast-enhanced images)

MRI showed pituitary stalk thickening in all seven patients, which was moderate (3.0–7 mm) in three patients including one child, and marked (>7 mm) in four patients including two children. In two cases, the lesion extended to the hypothalamus, and in the other two cases, the anterior pituitary was clearly involved. Optic chiasma involvement was not observed. All patients received gadolinium-enhanced MRI scans, and all showed enhancement (Fig. 1a, b). There were no hyperintense signals on T1WI in the posterior pituitary lobe in all seven patients. PET/CT was carried in two patients, and high uptake was observed in the pituitary stalk. The detailed results of the patients’ MRI scans of the HP region are listed in Table 2.

**Table 2:** Magnetic resonance imaging findings in the hypothalamic-pituitary regions of seven patients with hypothalamic-pituitary Langerhans cell histiocytosis

No.	Post signal	AP	WPS (mm)	HI	Details of the lesions					Changes during follow-up
					Number	Signal intensity on T1WI	Signal intensity on T2WI	Enhancement	Border	
1	Absent	Normal	7.1	Absent	Single	Isointensity	Isointensity	Homogeneous	Relatively clear	Regression
2	Absent	Normal	7.0	Absent	Single	Isointensity	Isointensity	Homogeneous	Relatively clear	Stable
3	Absent	Normal	6.6	Present	Single	Isointensity	Isointensity	Homogeneous	Irregular	Increased, PS enlargement
4	Absent	Enlarged	5	Absent	Single	Isointensity	Isointensity	Homogeneous	Obscure	Not available
5	Absent	Normal	8.5	Present	Single	Isointensity	Isointensity	Homogeneous	Relatively clear	Regression
6	Absent	Enlarged	6.6	Absent	Single	Slightly Hypointense	Slightly Hyperintense	Heterogeneous	Obscure	Stable
7	Absent	Normal	9	Absent	Single	Isointensity	Isointensity	Homogeneous	Relatively clear	Regression

*AP* anterior pituitary, *WPS* width of the pituitary stalk, *HI* hypothalamus involvement, *PI* pituitary involvement

### Anterior pituitary function

Four patients had anterior pituitary deficiency. Three of the four patients showed whole anterior pituitary deficiency, and the other patient had GH deficiency (GHD) and hypogonadism before diagnosis but developed whole anterior pituitary deficiency in the following 4 months. Three patients had normal anterior pituitary function before diagnosis, and anterior pituitary function was also normal in two patients in the following 2 and 5 months, respectively. Data on the anterior pituitary function of the third patient was absent (details in Table 3).

**Table 3:** Anterior pituitary function at presentation and during follow-up

No.	At presentation												Follow-up
	AP deficiency	GH (ng/mL, <2.5)	IGF1 (ng/mL, 74–388)	ACTH (pg/mL, 0–46)	Cortisol (μg/dL, 4–25)	LH (IU/L, 1.2–58.64)	FSH (IU/L, 1.27–113.59)	PRL (ng/mL, 2.74–26.72)	E2 (pg/mL, 20–273)	T (ng/dL, 0.1–7.8)	TSH (μIU/mL,0.38–4.34)	FT4 (ng/dL,0.81–1.89)	AP deficiency
1	Panhypopituitary	0.2	25	15.29	0.81	0	0.2	15.84	10.4	11.2	0.202	0.56	Panhypopituitary
2	Not impaired	0.5	129	28. 6	13.51	3.12	4.91	37.1	44	0.19	1.54	1.22	Not impaired
3	GH, LH-FSH deficiency	<0.05	72	26.3	12.05	0.43	0.36	17.39	25	<0.1	8.485	1.01	Panhypopituitary
4	Not impaired	1.4	198	30.6	12.9	2.63	1.0	15.6	11.8	360.8	2.662	1.380	NA
5	Panhypopituitary	<0.05	44	7.6	0.74	0	0.3	11.7	10.2	5.2	2.539	0.60	Panhypopituitary
6	Not impaired	0.2	381	51.3	13.65	4.7	4.42	18.14	56	2.11	2.581	1.333	Not impaired
7	Panhypopituitary	0.12	68	7.6	0.2	5.2	1.67	51.22	0.09	0.73	0.26	0.77	Panhypopituitary

*AP* anterior pituitary, *GH* growth hormone, *IGF1* insulin-like growth factor-1, *ACTH* adrenocorticotropic hormone, *LH* luteinizing hormone, *FSH* follicle-stimulating hormone, *PRL* prolactin, *E2* oestradiol, *T* testosterone, *TSH* thyroid-stimulating hormone, *FT4* free thyroxine

### Pathological and immunohistochemical findings

**Fig. 2:**
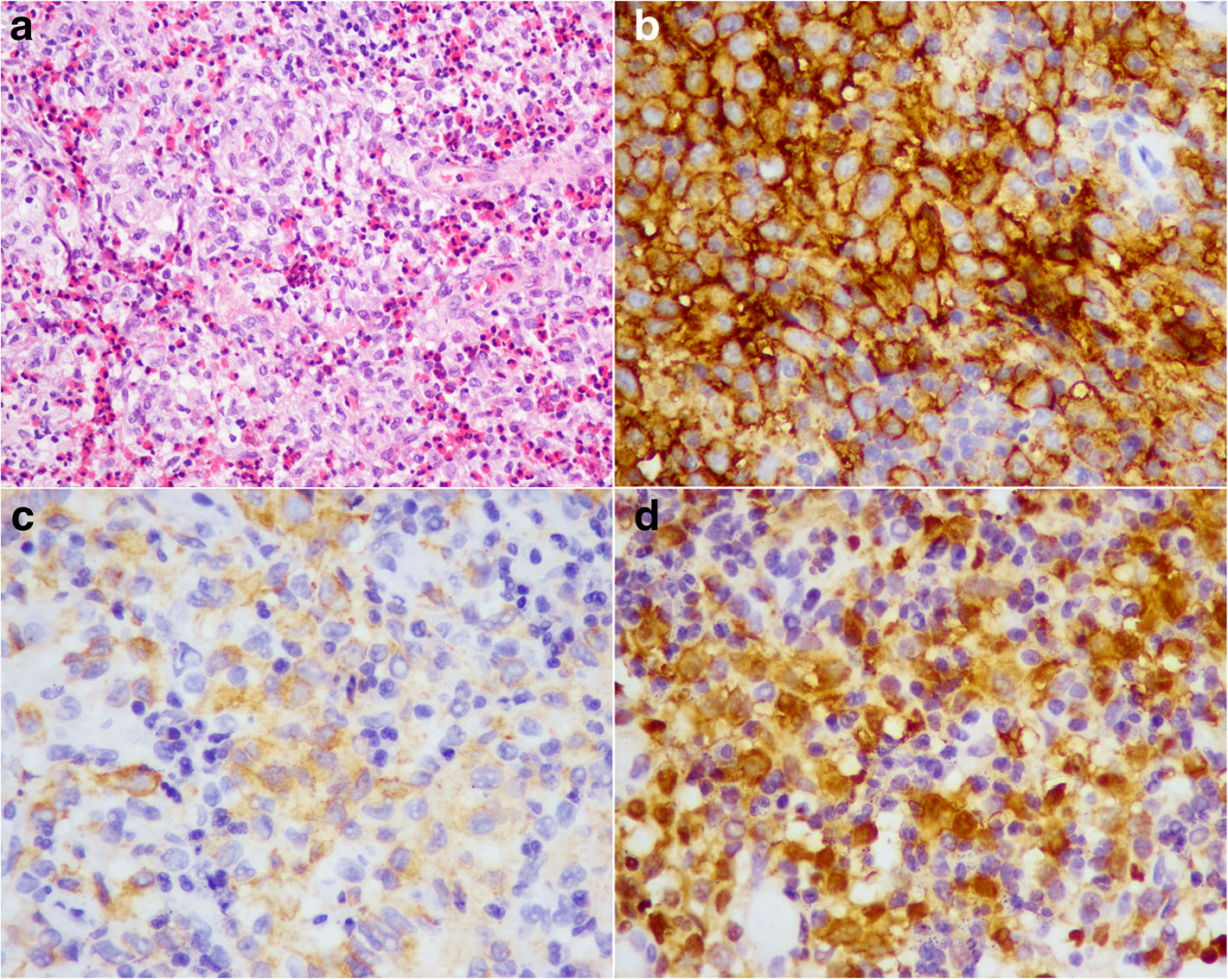
**a**. The lesion was predominantly composed of histiocytic cells and eosinophils (haematoxylin and eosin stain, 200×). **b**. The histiocytic cells were immunopositive for CD1a (400×). **c**. The histiocytic cells were immunopositive for Langerin (400×). **d**. The histiocytic cells were immunopositive for S-100 (400×)

Surgery of the HP region was performed in all cases and was conclusive in all cases. Six patients received a biopsy, and one patient received a complete mass resection. Macroscopically, the maximum diameter of the samples ranged from 3 to 10 mm, and all tissues were yellow or white. Microscopically, all lesions consisted of accumulated mononuclear histiocytic cells. The lesions in all seven cases were predominantly composed of histiocytic cells and a variable fraction of eosinophils (Fig. 2a). The nuclei of the histiocytic cells were characteristic slightly eccentric, ovoid, or reniform, with linear grooves and inconspicuous nucleoli. The cytoplasm was abundant and pale to eosinophilic. Mitotic figures were seldom observed, and no necrosis was found in all cases. Eosinophils infiltration was easily observed. Touton giant cells were absent in all cases. Immunohistochemical staining for CD1a (Fig. 2b), Langerin (Fig. 2c), S-100 (Fig. 2d), and CD68 was positive in the histiocytic cells of all 7 LCH cases (7/7).

### BRAF V600E gene status

**Fig. 3:**
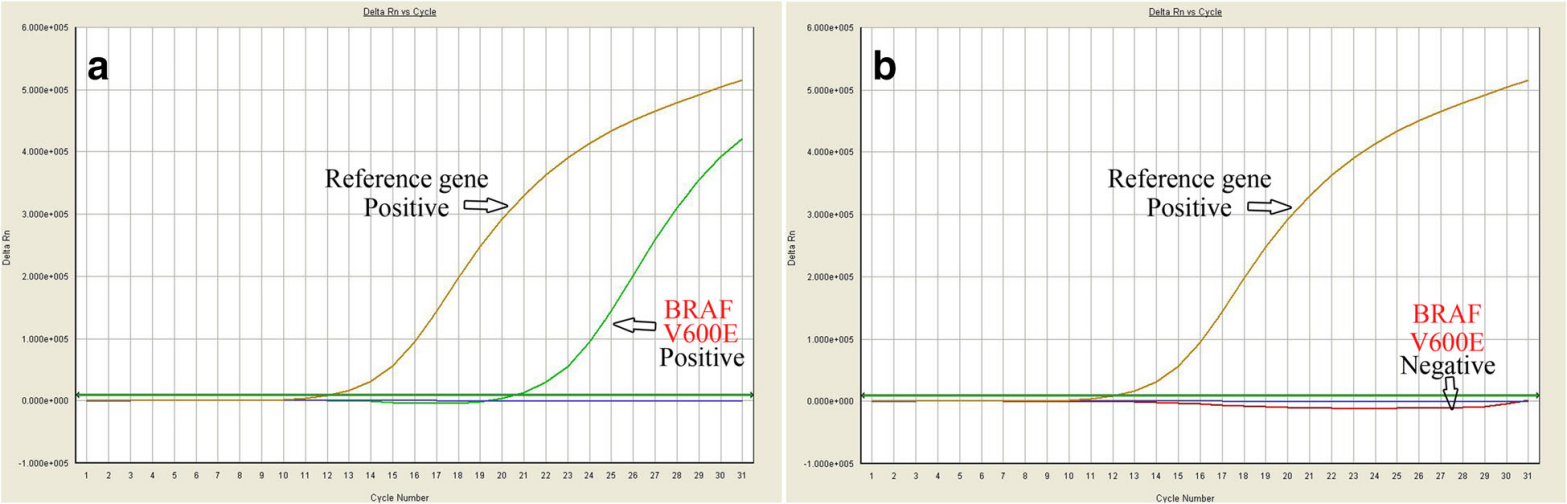
**a**. The point mutation V600E of BRAF was identified in case 2 by the QPCR method. **b**. No BRAF mutation was found in case 5 by the QPCR method

BRAF V600E mutations were found in three cases (3/6) of HPLCH using QPCR in 6 successfully amplified cases (Fig. 3a, b).

### Treatment and outcomes

Six cases had follow-up data (range 2–74 months, details in Table 3). All patients received desmopressin acetate and high-dose corticosteroid therapy. In four patients with anterior pituitary deficiency, three were also treated with Letrox, and one patient received testosterone replacement therapy. Two patients with whole anterior pituitary deficiency received focal radiotherapy of the HP regions (total dose: 30.5 Gy). One patient underwent surgical resection after CyberKnife radiosurgery (details not available) in another hospital. No patients received chemotherapy. During the follow-up, no patients developed extrapituitary involvement, except one patient who died and had suspected liver involvement, but no direct proof, 74 months after resection. Among the five remaining patients, three patients survived with diseases, and two patients who received RT survived without diseases. One patient was lost to follow-up after biopsy.

## Discussion

Langerhans cell histiocytosis (LCH) is a rare heterogeneous histiocytic disorder, and the dominant sites of involvement are bone and adjacent soft tissue, liver, spleen and bone marrow [1, 2, 15]. The central nervous system (CNS) may also be involved [2, 21]. In patients with LCH in the HP region, most cases are extended from osseous foci in multifocal or disseminated LCH, but unifocal or multifocal infiltrates can occur primarily within or even restricted to the HP region and other sites, such as the infundibulum, optic chiasm, and choroid plexus [2–7, 22, 23]. However, isolated HPLCH without the involvement of other organs is a rare condition, and there are few reports on the various characteristics of this disease state, including the evaluation of pituitary function, especially anterior pituitary function, the BRAF^V600E^ mutation status, and other clinicopathological features. Here, we reviewed seven cases of isolated LCH limited to the HP region and investigated the clinicopathological characteristics, endocrine function changes, BRAF^V600E^ mutations and treatment of the lesions.

The aetiology of the histiocytic lesions is largely unknown [1, 2]. Although the underlying aetiology of the condition has long been enigmatic, recent studies have suggested that LCH is due to clonal neoplastic proliferation of myeloid-derived precursor cells, with a high frequency of somatic oncogenic BRAF^V600E^ mutations in 25–60 % of LCH patients [13–20]. In CNS, BRAF alterations are found in variable frequencies across a wide spectrum of diverse neoplasms, such as various glial and glioneuronal tumours, craniopharyngiomas, LCH and brain metastases [16]. No association between the BRAF^V600E^ mutation and survival has been found in LCH, but several case reports described an impressive response to a BRAF inhibitor in patients with BRAF V600E-mutated LCH [19]. As an independent condition, the data on the BRAF ^V600E^ mutation in isolated HPLCH is unclear, and one reason for this may be the low number of cases. In this report, we examined seven cases of isolated HPLCH, and we had sufficient quantities of tissue for mutation detection in six cases. The results of our study showed that BRAF^V600E^ was mutated in 50 % of patients with HPLCH, suggesting that the mutation rate in isolated HPLCH is consistent with the reported rates in LCH involving other sites. Because the follow-up time for some patients was short in our study, the relationship of BRAF^V600E^ mutation and survival was not analysed. Smoking is an important factor in primary LCH of the lung [17], but all seven patients had no history of smoking in our study, suggesting that smoking is not related to HPLCH.

Clinically, LCH can occur at any age but typically occurs in the paediatric population [1, 6, 10]. The most common neurological signs of LCH are CDI with or without associated signs of hypothalamic dysfunction, increased intracranial pressure, cranial nerve palsies, and seizures [2]. Isolated CDI may be an early diagnostic signature in patients with an abnormal lesion in the HP region and no past medical history, and the association of polyuria-polydipsia suggests that a complete endocrine evaluation and a meticulous MRI examination of the HP region should be performed [2, 4, 6–10]. Patients with unifocal disease are usually older children or adults [1]. In our cohort, four patients were children aged 9–15 years, and three patients were adults. Our study had slightly more children with isolated HPLCH than adults. There was no specific gender preference in our study, which had slightly more females than males. The most common sign was CDI (polyuria and polydipsia, 100 %), and other symptoms included secondary amenorrhoea (1/7) and growth retardation (1/7).

While CDI is the most common HP symptom, anterior pituitary deficiencies are less common. Several studies have shown that most patients with HP lesions had one or more anterior pituitary deficiencies, and hormone deficiencies may be present at diagnosis or appear gradually during the course of disease [2–8, 11]. Regular monitoring of these patients is recommended. Growth hormone deficiency is the most frequent disease-related anterior pituitary deficiency and is found in up to 50 % of patients, followed by gonadotrophin deficiency, but ACTH and TSH are relatively less common; moderately elevated PRL levels are infrequently found and are attributed to pituitary stalk involvement [8]. Established deficiencies almost never recover over time. In our cohort, more than a half of the patients with HPLCH had anterior pituitary deficiencies at diagnosis with a mean interval from the first symptoms to diagnosis of 42 months. Three patients were diagnosed with normal anterior pituitary functions, and the intervals from the first symptoms to diagnosis were less than 2 months for all of them. Our study suggested that the interval from the first symptoms to diagnosis may be related to the anterior pituitary function, and the lesions progressed with time. All patients had posterior pituitary deficiencies at diagnosis, and polyuria and polydipsia were the first symptom in all patients; thus, a MRI scan is necessary for patients presenting with polyuria and polydipsia.

MRI changes in patients with LCH of CNS include the following: 1) intracranial, extra-axial changes of the hypothalamic-pituitary region (50 %), meninges (29 %) or choroid plexus (6 %); 2) intracranial, intra-axial changes of white matter and gray matter (36 %); and 3) cerebral atrophy (8 %) [2]. There are several studies on the MRI changes in HPLCH, and pituitary stalk thickening can be observed in LCH in the HP regions [4, 6]. In our cohort, the MRI features of patients with HPLCH included the following characteristics: 1) a single lesion on the HP region and involvement of the pituitary stalk; 2) isointensity signals on both T1WI and T2WI in most cases; 3) homogeneous enhancement in most cases; 4) relatively clear borders in most cases. Although these are characteristic MRI findings, it is difficult to distinguish HPLCH from other diseases, such as germinoma or lymphocytic thyroiditis.

An accurate pathological diagnosis of LCH is necessary for further treatment and follow-up depending on the classic pathomorphological and immunohistochemical features. Recently, the use of neuroendoscopic biopsy for suprasellar masses has increased, and with this technique, it is possible to obtain an accurate pathological diagnosis [24], although sometimes the pathological diagnosis is difficult due to small specimens with prominent sampling artefacts and haemorrhage [25]. Distinguishing this condition from other diseases that can cause CDI, such as craniopharyngioma, germinoma, sarcoidosis, hypophysitis, and tuberculosis, is essential [2, 10, 26], and the pathological signature with both CD1a-positive, Langerin-positive, and S-100-positive infiltrating histiocytes and increased presentation of eosinophils has made the differential diagnosis relatively easy. In addition, differentiation from other non-Langerhans cell histiocytoses, such as Erdheim-Chester disease or Rosai-Dorfman disease, is also important. Although S-100 positive cells can be found in Erdheim-Chester disease (ECD) and Rosai-Dorfman disease, CD1a is always negative in the same histiocytes [1, 2]. Notably, the BRAF^V600E^ mutation was detected in more than 60 % of patients with ECD [27], but this mutation could not distinguish ECD from LCH because it was detected in approximately 25–60 % of the latter samples [13–20]. In our cohort, the seven isolated HPLCH cases that we assessed had been confirmed by pathomorphological changes and immunohistochemical findings during neuroendoscopic transnasal transsphenoidal surgery, including six cases by biopsy and one by resection, and all cases were simultaneously positive for CD1a, Langerin and S-100. In addition, we evaluated the status of other organs in all patients, which is required to exclude the multisystemic lesions, and there was no evidence of other system involvement in all patients.

The overall 5-year survival rate of LCH patients is 88 %. Patients with unifocal LCH have an excellent prognosis and a high long-term survival rate (99 %) and may spontaneously recover or require minimal treatment [1, 28]. In our cohort, one patient died of liver disease, but we had no evidence of liver involvement with LCH; the other six patients were still alive, and further follow-up should be performed. No lesions were spontaneously recovered in our study. The treatments for LCH involving the CNS, such as conservative treatment, surgical intervention, chemotherapy and radiotherapy, were dependent on the lesion location, size, number and symptoms, and high-dose corticosteroid therapy was also used to control the inflammatory process. Additionally, CyberKnife radiosurgery was performed in some patients [2, 21, 28–30]. However, the treatment of HPLCH patients is controversial because localized radiotherapy and chemotherapy were associated with tumour regression and stabilization but did not appear to prevent the evolution or deterioration of LCH-associated endocrine deficiency [3–7, 30]. In our cohort, although remission was observed in the lesions of two patients after radiotherapy (30.5 Gy) and the lesion of another patient who received surgical resection after CyberKnife radiosurgery failure, the patients’ quality of life was irreversibly impaired because they had irreversible permanent sequelae, panpituitary hormone deficiency, and needed to receive goal-directed hormone replacement therapy and monitor hormone level changes permanently. The anterior pituitary function of another patient who received high-dose corticosteroid therapy only also progressed from GH and LH-FSH deficiency to panpituitary hormone deficiency. Our study suggested that the treatment of patients with HPLCH is a difficult problem, and the deterioration of the endocrine function appeared to be unrelated to lesion regression; thus, diagnosis and treatment as early as possible are essential. In addition, monitoring the whole body to assess multisystemic involvement is also very important. Moreover, for patients with BRAF^V600E^ mutations, new targeted therapies, such as vemurafenib, may be used because the results have been encouraging in select progressive patients with LCH [17, 31]. Recently, Nakagawa et al. [12] reported two relapsed females with LCH limited to the pituitary stalk and found that chemotherapy with cytarabine may be useful for preventing anterior pituitary hormone deficiency and neurodegenerative disease for new-onset CDI with LCH, but the further studies are needed to confirm these results.

Our study has some limitations as it is based on a small group of selected patients due to the low incidence of this condition. Notably, the follow-up time is short in our study because neuroendoscopic biopsy is a new technique in recent years, and it will be necessary to follow-up the patients over a longer period of time.

## Conclusions

Our study indicated that some cases of isolated LCH involve only the hypothalamic-pituitary region, and all patients had CDI as the earliest symptom, with more than half having anterior pituitary deficiencies. The BRAF^V600E^ mutation is a common genetic change in HPLCH patients. The treatment of HPLCH patients is difficult, and the loss of endocrine function is irreversible in most cases. For patients with the BRAF^V600E^ mutation, new targeted therapy may be used when the lesion is progressing.

## Abbreviations

ACTH: Adrenocorticotropic hormone
AP: Anterior pituitary
CDI: Central diabetes insipidus
CNS: Central nervous system
CT: Computed tomography
E2: Oestradiol
ECD: Erdheim-Chester disease
FSH: Follicle-stimulating hormone
FSH: Follicle-stimulating hormone
FT4: Free thyroxine
GH: Growth hormone
GHD: Growth hormone deficiency
HP: Hypothalamic-pituitary
IGF1: Insulin-like growth factor-1
LCH: Langerhans cell histiocytosis
LCH: Langerhans cell histiocytosis
LH: Luteinizing hormone
LH: Luteinizing hormone
MRI: Magnetic resonance imaging
PRL: Prolactin
QPCR: Quantitative polymerase chain reaction
RT: Radiotherapy
T: Testosterone
TSH: Thyroid-stimulating hormone
